# Validity and reproducibility of a food frequency questionnaire assessing food group intake in the PERSIAN Cohort Study

**DOI:** 10.3389/fnut.2023.1059870

**Published:** 2023-08-04

**Authors:** Sareh Eghtesad, Azita Hekmatdoost, Elnaz Faramarzi, Reza Homayounfar, Maryam Sharafkhah, Hamid Hakimi, Ali Dehghani, Mahmood Moosazadeh, Zinat Mortazavi, Yahya Pasdar, Hossein Poustchi, Walter C. Willett, Reza Malekzadeh

**Affiliations:** ^1^Liver and Pancreatobiliary Diseases Research Center, Digestive Diseases Research Institute, Tehran University of Medical Sciences, Tehran, Iran; ^2^Department of Clinical Nutrition and Dietetics, National Nutrition and Food Technology Research Institute, Shahid Beheshti University of Medical Sciences, Tehran, Iran; ^3^Liver and Gastrointestinal Diseases Research Center, Tabriz University of Medical Sciences, Tabriz, Iran; ^4^Noncommunicable Diseases Research Center, Fasa University of Medical Sciences, Fasa, Iran; ^5^Faculty of Nutrition and Food Technology, National Nutrition and Food Technology Research Institute, Shahid Beheshti University of Medical Sciences, Tehran, Iran; ^6^Immunology of Infectious Diseases Research Center, Rafsanjan University of Medical Sciences, Rafsanjan, Iran; ^7^Centre for Healthcare Data Modeling, School of Public Health, Shahid Sadoughi University of Medical Sciences, Yazd, Iran; ^8^Gastrointestinal Cancer Research Center, Non-communicable Diseases Institute, Mazandaran University of Medical Sciences, Sari, Iran; ^9^Health Promotion Research Center, Zahedan University of Medical Sciences, Zahedan, Iran; ^10^Nutritional Sciences Department, Research Center for Environmental Determinants of Health (RCEDH), Kermanshah University of Medical Sciences, Kermanshah, Iran; ^11^Department of Nutrition, Harvard T. H. School of Public Health, Boston, MA, United States; ^12^Department of Epidemiology, Harvard T. H. School of Public Health, Boston, MA, United States; ^13^Digestive Diseases Research Center, Digestive Diseases Research Institute, Tehran University of Medical Sciences, Tehran, Iran

**Keywords:** food frequency questionnaire, FFQ, PERSIAN cohort, validity, reproducibility

## Abstract

**Purpose:**

A semi-quantitative food frequency questionnaire (FFQ) was developed for use in the Prospective Epidemiological Research Studies in IrAN (PERSIAN Cohort), investigating non-communicable disease risk factors. This study aimed to assess the validity and reproducibility of this FFQ, through food group intake.

**Methods:**

Participants, recruited from seven PERSIAN cohort centers, completed the FFQ at the beginning of the study (FFQ1) and at the end (FFQ2), with a 12-month interval in between, during which two 24-h dietary recalls (24 h) were completed each month. Correlation coefficients of the median intake of food groups recorded by the FFQs were compared to those of the 24 h to assess validity, and the two FFQs were compared to assess reproducibility of findings.

**Results:**

Overall, data from 978 participants were included in this validation analysis. Of the 26 food groups assessed, *Tea*, *Sugars*, *Whole/Refined Grains*, and *Solid Fats*/*Oils*, had the strongest correlations (0.6–0.79), while *Red Meat, Chicken* and *Eggs* showed moderate correlations (0.42–0.59). The weakest correlations observed belonged to *Fresh fruit Juice* and *Other Meats* (0.23–0.32). Reproducibility was assessed among those who completed both FFQ1 and FFQ2 (*n* = 848), revealing moderate to strong correlations in all food groups, ranging from 0.42 in *Legumes* to 0.72 in both *Sugar* and *Sweetened Drinks.*

**Conclusion:**

The PERSIAN Cohort FFQ is appropriate to rank individuals based on food group intake.

## Introduction

The Prospective Epidemiological Research Studies in IrAN (PERSIAN Cohort) is the largest cohort study in Iran, aiming to investigate risk factors of common non-communicable diseases (NCDs) in different geographical areas and among various ethnic populations of Iran. Among many questionnaires completed for participants to obtain baseline information on lifestyle, environmental, and social exposures, a semi-quantitative food frequency questionnaire (FFQ) was developed for use in the PERSIAN Cohort Study, to assess diet’s role in NCD development.

Two semi-quantitative FFQs had been previously developed and validated in Iran, but were of limited use in the PERSIAN Cohort Study, because they were validated in specific populations, not best depicting the PERSIAN Cohort population. The FFQ used in the Golestan Cohort Study (GCS) was validated among the Turkmen ethnic population (2% of Iran’s total population), who have specific dietary habits and local food items, while the questionnaire used in the Tehran Lipid and Glucose Study (TLGS), was validated among the capital city’s population, whose dietary habits are again different from those of many smaller cities and rural areas included in the PERSIAN Cohort Study (1, 2). Besides the population differences, both of these FFQs were long, with the GCS FFQ including 150 and the TLGS including 168 food items. Given that multiple questionnaires are completed for each individual who enrolls in the PERSIAN Cohort, a shorter FFQ was desired, to reduce participant fatigue, which can subsequently affect response accuracy. A simplified FFQ, on the other hand, including 48 items was also validated for use in the Isfahan Healthy Heart Program (IHHP). The items in this questionnaire were chosen with a focus on foods affecting cardiovascular diseases and thus, it was not comprehensive enough to be used for assessing diet-NCD relationships in the PERSIAN Cohort population (3).

The aim in the development of the PERSIAN Cohort FFQ was therefore, to develop a comprehensive, yet shorter FFQ for possibility of use in different populations of Iran with varying dietary habits. To assess the validity and reproducibility of this questionnaire, a multi-center study was designed and executed in seven different PERSIAN Cohort centers, in order to better capture the dietary variations of the PERSIAN Cohort participants.

Given that individual nutrients, foods, food groups and dietary patterns can influence disease development, FFQ validation at all levels is recommended (4, 5). In this manuscript, we report the validity and reproducibility of the PERSIAN Cohort FFQ in assessing *food group* intake.

## Materials and methods

We conducted this study, parallel to the pilot phase of the PERSIAN Cohort Study, the methodology and rationale of which have been previously published (6, 7). Briefly, PERSIAN started in 2014 in 18 locations of Iran. Individuals aged 35–70 years were invited to participate and those who agreed, reported to the cohort center on their appointment date, when laboratory tests, anthropometric measurements and interviewer-administered questionnaires were completed, including an FFQ. All participants are currently being followed annually to record the occurrence of common NCDs or death.

### Study participants

We chose this study’s participants from those enrolling in the pilot phase of the PERSIAN Cohort study. Our inclusion criteria parallels that of PERSIAN’s, which enrolled men and women of Iranian descent, who were 35–70 years of age, and who resided in the designated cohort areas. The only exclusion included having a physical or psychological disability that hinders participation in the study by interfering with accurate data collection (6).

Given that the pilot phase at different PERSIAN Cohort centers started at various times, this study stretched over approximately three years, from January 2015 to November 2017. During this time, 1,260 individuals who enrolled in the PERSIAN Cohort at the Fasa, Rafsanjan, Azar, Yazd, Ravansar, Zahedan, and Tabari cohort centers (180 from each center), were also invited to participate in this validation study. Of these individuals, 1,097 agreed to participate. Sample collection for the validation study relied on invitations in the main cohort and when the desired sample size was reached at each center, enrollment ceased. These seven cohort centers were chosen in order to include major ethnic populations of Iran as well as geographical areas, with varying lifestyles and eating habits. This study was approved by the ethics committee of the Digestive Diseases Research Institute, Tehran University of Medical Sciences (IR.TUMS.DDRI.REC.1398.001). Written informed consent was obtained from all participants.

### FFQ development and completion

The PERSIAN Cohort FFQ was developed by modifying the GCS FFQ, which included 150 single food items, about 90 of which were common foods used throughout Iran and 10, local to Golestan province (1). The remaining items were either variations of the same foods included, or foods neither local to Golestan, nor commonly used elsewhere in Iran. We also evaluated foods included in the TLGS FFQ and finally selected 113 food items categorized in 9 major groups, as the *standard FFQ items* (2). These items were chosen by nutrition experts, and based on their frequency of use in the Iranian diet, their energy-contribution, as well as access to the items throughout Iran. Local experts at each cohort center were also consulted and if food items not included in the standard items were identified that were either used frequently in that population, or were nutrient and/or calorie-dense, these items were also added to the FFQ for that center only, as *local food items.* These mostly consisted of local breads, sweets, or few fruits and vegetables and varied between five to ten items per center. In some centers, the interviewers were instructed to add the amount of a specific *local item* consumed to one of the *standard items*, if the two items were very close in composition. In many cases however, to limit data collection mistakes, information on the *local food items* were recorded as separate items and later equated to the *standard items* by nutritionists based on their major ingredients.

We chose to include food items in this FFQ, rather than dishes, because while many dishes in the Persian cuisine are well-known and made throughout Iran, the ingredients used in those dishes sometimes differs from one area to another. Also, Persian dishes are very ingredient-rich and individual variations and preferences put into recipes also make a dish-based FFQ that is reflective of all the variations, difficult to design and analyze.

Our FFQ was designed as a semi-quantitative, interviewer-administered questionnaire, enquiring about individuals’ usual intake of each food item over the year prior to the interview date. Participants reported their daily, weekly, monthly or yearly use of each item, as well as the portion consumed each time, based on portion sizes pertaining to each item. Actual dish, cups and utensils, as well as several portion size models were used for a more precise portion size estimation. In addition, a 64-picture album including standard portions for selected items was used whenever needed (8). All tools were centrally purchased and distributed to cohort centers to ensure consistency and all interviewers were trained by the same person, using the same study protocol.

Given that all individuals aged 35–70 years were invited to participate in the cohort study, most participants enrolled along with and on the same day as other family members (spouses or parents). While all procedures were completed for each individual separately, the FFQ of spouses were completed at the same time and by the same interviewer, since women predominantly cook in the Iranian culture and information regarding many ingredients used in cooking is not well-known by men. Women reported the frequency of use and overall amount of these items they typically use in cooking, and then each person’s share was determined and recorded in their questionnaire. If individuals did not enroll with their spouses or were single, information on these items was asked from pertinent family members, by phone.

### Reference method and data collection timeline

The 24-h dietary recall (24 h) method was used as the reference method for FFQ validation. These recalls were also interviewer-administered and were completed in person. The United States Department of Agriculture (USDA) multiple-pass method was used to complete the 24 h (9). The same tools used to record FFQ portion sizes were also used when obtaining the 24 h and again pertinent family members were consulted in the completion of the 24 h, if the participant was not involved in cooking.

Upon entering the validation study, an FFQ was completed for each participant (FFQ1). Then, 24 h were completed twice monthly for 12 months, followed by another FFQ at the end of the study (FFQ2). To assess validity, data obtained from the 24 h were compared to those recorded by the FFQs and the two FFQs were compared in the reproducibility assessment of the study.

### Missing data

Missing data was not observed in the FFQs, since all questionnaires were completed on a smart electronic questionnaire that alarmed missing values upon completion. Missing an entire 24 h or FFQ2 did on the other hand occur, as sometimes participants did not meet their scheduled appointment to complete the questionnaires or were no longer interested to cooperate. When a visit to the cohort center was not possible, interviewers were instructed to complete the 24 h by phone to limit missing 24 h. Although two 24 h were to be obtained from each participant each month, when it was not possible to obtain two, having one recall per month was also considered adequate. However, participants with either more than 12 recalls missing, or those missing all 24 h in one season, were excluded from the analysis.

As for FFQ2, participants were invited to the cohort center three times to complete the questionnaire at the end of the study, and afterwards were considered missing and were excluded from any analysis requiring data from FFQ2.

### Data processing

Frequency data obtained for each food item on the FFQs were converted to daily intake, then multiplied by the weight (in grams) of the portion size consumed each time to obtain the *grams consumed from each food item per day* (grams/day). For the 24 h, the grams/day was calculated by adding the amount of each food item consumed in all 24 h, then dividing the sum by the number of 24 h obtained.

The USDA Food Composition Tables (USDA-FCT) were used to obtain daily energy intake of food items (10). Standard, non-branded foods in the USDA-FCT, checked by four nutritionists to be the best equivalent of the Iranian food items in regards to ingredients and macronutrients were chosen for energy estimations. For several foods native to Iran, not included in the USDA-FCT, the weighted average of major ingredients was used to equate that food item. The *local food items* were also, as previously stated, equated to the *standard FFQ items*, based on their major ingredients.

For the purpose of the food group analysis, food items were first grouped based on the USDA MyPlate groups, then, further narrowed based on major and important ingredients. Total food group intake was obtained by adding the grams/day consumption of all food items within each group.

### Statistical analysis

Kolmogorov–Smirnov test and Q-Q normal plot were used to test the normality assumption for all food groups. Since the distribution of most food groups were skewed, medians with the first and third quartiles [interquartile range (IQR)] were used to describe the food group intakes in the questionnaires examined. Crude (C), energy-adjusted (EA) and de-attenuated energy-adjusted (DEA) Spearman’s rank correlation coefficients (SCC) were obtained to assess the validity of FFQ1 and FFQ2 relative to the 24 h. EA-SCC were calculated using the nutrient density approach (11). The DEA-SCC, which was corrected for intra-person variability in the 24 h, was calculated through the following formula:

energy−adjustedSCC×[1+λ/n]1/2

where n is the number of 24 h replicates (24 in this study), and λ is the ratio of within-person and between-person variance (4). Food groups were categorized into tertiles to examine agreement between the questionnaires. Agreement was described as the proportion of individuals classified in the same, adjacent and extreme categories.

To assess reproducibility, crude and energy-adjusted Intraclass Correlation Coefficients (C-ICC and EA-ICC, respectively) and their 95% confidence intervals (CI) were calculated between FFQ1 and FFQ2. Cross-classification analysis was also conducted. All statistical analyses were performed using the statistical software STATA 12 (StataCorp, College Station, TX, United States). *p* < 0.05 was considered as statistically significant for all tests.

## Results

**Figure 1:**
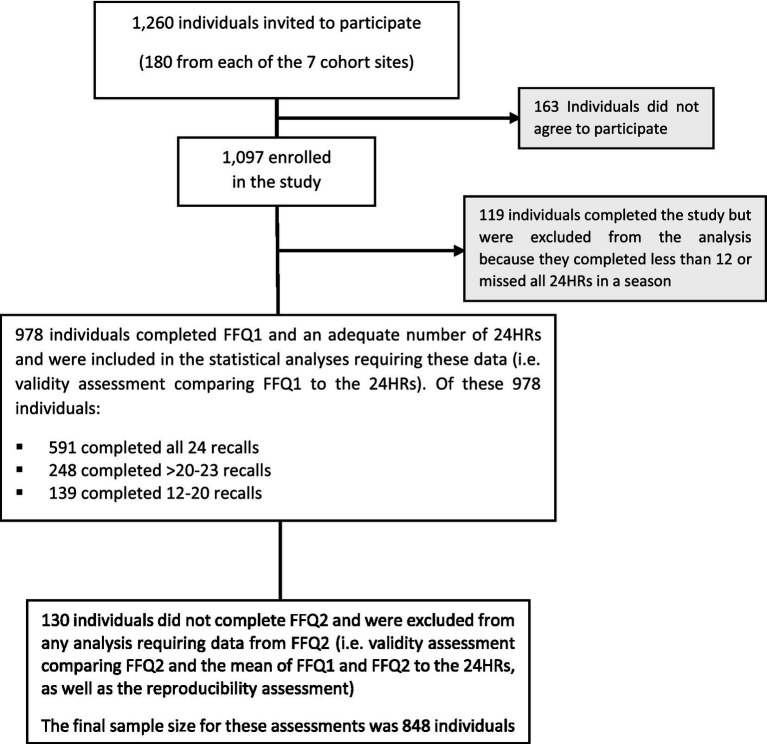
Participant recruitment and retention in the PERSIAN Cohort FFQ validation study.

A total of 1,097 individuals entered this study; 76.5% completed more than 20 recalls (53.9% completed all 24), while 10.8% completed less than 12 and were excluded from all analysis, leaving 978 individuals as the final study population (Figure 1). Age, gender and BMI of those excluded was not significantly different from the remaining participants (data not shown). Baseline characteristics of participants are shown in Table 1. Mean age was 46.6 ± 8.25 years and 58% were female. While over 90% of individuals had some formal education, 42.8% had only primary education or were illiterate.

**Table 1:** Baseline characteristics of the study population.

		Study participants *N* = 978
Age, Mean ± SD		46.6 ± 8.25
BMI, Mean ± SD		28.3 ± 7.95
Gender N (%)	Male	411 (42.0)
	Female	567 (58. 0)
Residency N (%)	Urban	794 (81.2)
	Rural	184 (18.8)
Education N (%)	Illiterate or primary	419 (42.8)
	Secondary or High school	429 (43.9)
	University Education	130 (13.3)

Comparing the median intake of food groups across the three questionnaires (Table 2), FFQ1 recorded higher intake in 14 of the 26 food groups while the 24 h recorded greater intake in 5 groups compared to the FFQs. The median intake of *Fresh Fruit Juice, Oils*, *Salty Snacks* and *Salt* were the same in FFQ1 and 2, while *Pizza* and *Olives* had zero median intake in all questionnaires.

**Table 2:** Median (IQR) intake of food groups in each questionnaire (g/day).

Food groups	FFQ1	FFQ2	24 h
Whole grains	16.2 (5.3–43.7)	12.5 (4.10–32.5)	17.1 (5.0–47.8)
Refined grains	384 (247–558)	379 (256–535)	322 (226–433)
Legumes	27.6 (16.2–46.3)	21.1(12.6–35.3)	24.4 (14.8–34.7)
Fish	3.7 (1.0–8.5)	3.0 (1.0–7.9)	1.7 (0–6.7)
Red meat	17.1 (7.8–34.2)	12.8 (5.3–26.7)	23.1 (13.8–37.7)
Processed meat	0 (0–0.4)	0 (0–0.2)	0 (0–0)
Chicken	17.1 (8.50–34.5)	17.1 (8.5–34.2)	24.1 (13.8–36.7)
Eggs	25.6 (8.50–34.2)	17.1(8.5–34.2)	18.8 (10.9–30.0)
Other meat	2.5 (1.1–5.8)	2.1 (0.8–4.8)	0 (0–5)
Pizza	0.1 (0–2.5)	0.2 (0–2.5)	0 (0–0)
Cheese	17.1 (8.5–30.0)	15.0 (8.5–30.0)	14.0 (8.1–21.2)
Dairy	237 (140–376)	228 (132–355)	125 (71.4–203)
Vegetables	459 (326–647)	392 (279–562)	289 (197–377)
Fresh fruit juice	1.3 (0–8.2)	1.3 (0–7.6)	0 (0–3.2)
Fruit	345 (198–561)	311 (165–523)	280 (192–391)
Dried fruit	12.8 (5.2–28.0)	12.5 (5.1–25.9)	6.3 (2.4–13.5)
Solid fats	8.5 (2.4–23.9)	7.0 (2.0–18.2)	7.8 (2.6–15.9)
Oils	6.0 (1.5–12)	6.0 (1.7–12.0)	5.0 (2.1–8.7)
Olives	0 (0–0.4)	0 (0–0.4)	0 (0–0)
Nuts and seeds	5.1 (1.8–11.3)	5.0 (2.0–10.2)	3.5 (1.1–7.5)
Sugar	27.8 (14.9–49.0)	23.9 (12.0–41.1)	20.0 (11.9–32.3)
Sweets	15.5 (7.8–30.9)	14.6 (7.0–27.7)	19.7 (9.0–33.5)
Sweetened drinks	24.9 (7.6–70.1)	19.2 (5.0–64.5)	26.8 (9.6–57.5)
Tea	690 (345–920)	575 (345–920)	518 (340–745)
Salty snacks	0.3 (0–1.6)	0.3 (0–1.3)	0 (0–0)
Salt	3.0 (2.25–4.5)	3.0 (2.3–4.0)	2.7 (1.9–3.1)

### Validity assessment

C-SCC, EA-SCC and DEA-SCC are shown in Table 3 comparing FFQ1, FFQ2 and mean of FFQ1 and 2 (FFQ1&2), vs. the 24 h. C-SCC and DEA-SCC ranged from 0.23 to 0.70, and 0.22 to 0.70, respectively, in comparing FFQ1 and 24 h, from 0.25 to 0.74, and 0.27 to 0.76 in FFQ2 vs. 24 h, and finally from 0.28 to 0.79 and 0.30 to 0.79 when the mean of FFQ1&2 and 24 h were compared, respectively.

**Table 3:** Crude, energy-adjusted and de-attenuated energy-adjusted Spearman correlation coefficients comparing FFQ1, FFQ2 and mean of FFQ1 and FFQ2 with the 24 h.

Food groups	FFQ1 vs. 24 h (*N* = 978)			FFQ2 vs. 24 h (*N* = 848)			FFQ1&2 vs. 24 h (*N* = 848)		
	Crude	Energy-adjusted	De-attenuated energy -adjusted	Crude	Energy-adjusted	De-attenuated energy-adjusted	Crude	Energy-Adjusted	De-attenuated energy-adjusted
Whole grains	0.53	0.51	0.68	0.53	0.52	0.69	0.53	0.51	0.68
Refined grains	0.70	0.54	0.65	0.67	0.50	0.60	0.74	0.56	0.67
Legumes	0.27	0.23	0.25	0.37	0.33	0.36	0.36	0.32	0.35
Fish	0.31	0.31	0.35	0.36	0.37	0.42	0.37	0.37	0.42
Red meat	0.48	0.44	0.53	0.53	0.49	0.59	0.53	0.49	0.59
Processed meat	0.38	0.38	0.43	0.39	0.39	0.45	0.41	0.41	0.47
Chicken	0.46	0.37	0.42	0.53	0.45	0.51	0.56	0.47	0.54
Eggs	0.45	0.40	0.46	0.48	0.43	0.49	0.52	0.49	0.56
Other meat	0.23	0.21	0.23	0.30	0.28	0.31	0.28	0.27	0.30
Pizza	0.26	0.27	0.31	0.29	0.30	0.35	0.29	0.30	0.35
Cheese	0.48	0.46	0.51	0.59	0.57	0.63	0.59	0.58	0.64
Dairy	0.51	0.46	0.56	0.59	0.55	0.66	0.63	0.59	0.71
Vegetables	0.52	0.46	0.55	0.53	0.56	0.67	0.61	0.59	0.71
Fresh fruit juice	0.26	0.27	0.29	0.25	0.25	0.27	0.29	0.30	0.32
Fruit	0.43	0.45	0.56	0.45	0.50	0.62	0.48	0.53	0.66
Dried fruit	0.39	0.42	0.44	0.41	0.43	0.45	0.45	0.48	0.50
Solid fats	0.61	0.58	0.65	0.70	0.68	0.76	0.73	0.70	0.78
Oils	0.55	0.57	0.65	0.67	0.67	0.76	0.66	0.68	0.77
Olives	0.35	0.35	0.36	0.42	0.43	0.44	0.41	0.42	0.43
Nuts and seeds	0.55	0.52	0.60	0.50	0.46	0.53	0.55	0.52	0.60
Sugar	0.67	0.63	0.70	0.74	0.66	0.73	0.76	0.71	0.79
Sweets	0.39	0.43	0.52	0.36	0.39	0.47	0.39	0.43	0.52
Sweetened Drinks	0.52	0.51	0.65	0.56	0.54	0.69	0.56	0.55	0.70
Tea	0.69	0.63	0.69	0.74	0.66	0.73	0.79	0.72	0.79
Salty snacks	0.32	0.32	0.34	0.31	0.31	0.33	0.34	0.33	0.35
Salt	0.36	0.35	0.37	0.40	0.41	0.43	0.46	0.45	0.48

At least seven groups had strong DEA-SCC (>0.6) in all three comparisons, including *Refined Grains*, *Solid Fats* and *Oils*. The *Fruits, Vegetables, Cheese,* and *Dairy* groups had moderate correlations (0.3–0.6) when FFQ1 was compared to the 24 h, and strong correlations with FFQ2 and FFQ1&2. *Red Meat*, *Chicken* and *Eggs* showed moderate correlations in all three comparisons. *Legumes* had a weak DEA-SCC (<0.3) when FFQ 1 and the 24 h were compared, but this group had moderate correlations in the other comparisons made. *Fresh fruit juice* and *Other Meats* showed weak correlations in two of the three comparisons.

Gender-specific SSC comparing the various questionnaires were also calculated (Supplementary Tables 1, 2). Correlation values observed in men and women for the various food groups, as well as patterns of food groups having strong and weak SCC, were similar to those observed for the entire study population.

On average, 54.3% [median (IQR): 50.2% (46.7 to 53.6%)], 51.6% [median (IQR): 50.7% (47.2 to 55.3%)], and 51.7% [median (IQR): 51.6% (47.3 to 54.3)] of participants were correctly classified into the same tertiles for all food groups in FFQ1 vs. 24 h, FFQ2 vs. 24 h, and mean of FFQ1&2 vs. 24 h, respectively (Table 4). The highest mismatch occurred for *Pizza*, in all comparisons [28.3% (FFQ1), 27.3% (FFQ2), 26.7% (FFQ1&2)], then for *Fresh Fruit Juice*, *Processed Meat*, *Olives* and *Salty Snacks*, with about one in four individuals being misclassified in these groups.

**Table 4:** Percent agreement for tertiles between FFQ1, FFQ2 and Mean of FFQ1&2 with 24 h.

Food groups	FFQ1 (%)			FFQ2 (%)			FFQ1&2 (%)		
	Same	Adjacent	Extreme	Same	Adjacent	Extreme	Same	Adjacent	Extreme
Whole grains	50.2	40.8	9.0	53.0	38.5	8.5	52.1	39.3	8.6
Refined grains	52.9	37.8	9.3	50.7	40.3	9.0	51.8	41.1	7.1
Legumes	40.3	43.2	16.5	43.5	42.9	13.7	42.4	43.2	14.4
Fish	45.4	38.2	16.4	47.2	38.7	14.2	46.6	38.9	14.5
Red meat	48.1	41.6	10.3	50.7	40.1	9.1	52.2	39.5	8.3
Processed meat	73.5	0	26.5	75.1	0	24.9	66.7	9.7	23.5
Chicken	46.7	40.1	13.3	49.1	40.5	10.3	51.4	39	9.6
Eggs	44.9	44.1	11.0	47.7	42.1	10.2	49.8	41.1	9.1
Other meat	40.2	37.1	22.7	42.2	38.5	19.2	42.9	37.4	19.7
Pizza	55.7	16.0	28.3	55.3	17.4	27.3	46.6	26.7	26.7
Cheese	48.8	41.3	10.0	54.8	38.3	6.9	54.3	39.4	6.3
Dairy	48.6	42.2	9.2	53.8	38.4	7.8	54.9	39.1	6.0
Vegetables	49.7	40.8	9.4	50.6	43.5	5.9	53.7	39.9	6.4
Fresh fruit juice	52.2	21.2	26.6	48.6	24.6	26.7	42.5	33.7	23.8
Fruit	50.2	39.7	10.2	51.4	39.5	9.1	51.8	39.3	8.9
Dried fruit	46.8	43.1	10.2	46.3	42.7	11.0	50.1	40.4	9.5
Solid fats	54.0	40.0	6.0	59.0	37.1	3.9	60.8	36.4	2.8
Oils	53.6	39.6	6.8	61.5	34.0	4.5	58.5	38.1	3.4
Olives	60.1	14.1	25.8	56.0	20.8	23.2	47.8	29.8	22.4
Nuts and seeds	53.4	38.1	8.5	48.4	40.9	10.7	50.7	40.6	8.7
Sugar	56.8	37.3	5.9	59.9	33.6	6.5	60.8	35.0	4.2
Sweets	47.1	42.3	10.6	45.0	43.3	11.7	47.3	42.7	10.0
Sweetened drinks	53.0	38.1	8.9	51.3	40.9	7.8	52.2	39.7	8.1
Tea	56.0	37.5	6.5	57.8	37.5	4.7	63.3	33.3	3.4
Salty snacks	50.6	24.3	25.1	45.0	29.8	25.2	42.9	33.0	24.1
Salt	45.8	41.4	12.8	46.8	41.3	11.9	50.3	37.9	11.8

### Reproducibility assessment

Of the 978 study participants, 848 (87%) completed FFQ2 and were included in the reliability assessment. Crude and energy-adjusted ICC (95% CI) for food group intake between the two FFQs are shown in Table 5. The C-ICC ranged from 0.4 (*Fresh fruit juice*) to 0.77 (*Refined grains*) and the EA-ICC from 0.42 (*Legumes*) to 0.72 (both *Sugar* and *Sweetened Drinks*). Strong correlations (>0.6) were observed in half of the 26 food groups, and moderate correlations (0.3–0.6) in the other half. *Same* category agreement ranged from 46.3 to 76%, averaging 54.6% of participants [median (IQR): 54.6 (51.5–57%)]. Gender-specific reproducibility also yielded similar results as that of the entire population (Supplementary Table 3).

**Table 5:** Reproducibility assessed by intraclass correlation coefficients (ICC) comparing FFQ1 and FFQ2 (*N* = 848).

Food groups	ICC (95% CI)		Agreement (%)		
	Crude	Energy-adjusted	Same	Adjacent	Extreme
Whole grains	0.63 (0.58–0.68)	0.65 (0.6–0.69)	53.4	38.6	8.0
Refined grains	0.77 (0.73–0.8)	0.68 (0.64–0.73)	50.2	41.4	8.4
Legumes	0.44 (0.36–0.51)	0.42 (0.34–0.49)	46.3	41.8	11.9
Fish	0.57 (0.51–0.62)	0.53 (0.46–0.59)	55.7	35.9	8.4
Red meat	0.61 (0.55–0.66)	0.61 (0.55–0.66)	54.3	37.8	7.9
Processed meat	0.53 (0.46–0.59)	0.61 (0.55–0.66)	76.0	0	24.0
Chicken	0.71 (0.67–0.75)	0.65 (0.6–0.69)	51.5	39.5	9.0
Eggs	0.54 (0.48–0.6)	0.53 (0.47–0.59)	47.7	41.6	10.7
Other meat	0.62 (0.57–0.67)	0.54 (0.48–0.6)	53.8	37.8	8.4
Pizza	0.57 (0.51–0.62)	0.57 (0.51–0.63)	69.7	22.4	7.9
Cheese	0.53 (0.47–0.59)	0.59 (0.53–0.64)	52.7	36.3	11.0
Dairy	0.69 (0.65–0.73)	0.66 (0.62–0.71)	51.1	40.7	8.1
Vegetables	0.64 (0.59–0.69)	0.6 (0.55–0.65)	52.7	38.5	8.9
Fresh fruit juice	0.4 (0.31–0.47)	0.51 (0.43–0.57)	55.4	31.3	13.3
Fruit	0.67 (0.62–0.71)	0.68 (0.64–0.72)	56.0	37.1	7.0
Dried fruit	0.61 (0.56–0.66)	0.55 (0.48–0.6)	50.3	39.3	10.4
Solid fats	0.67 (0.63–0.71)	0.69 (0.65–0.73)	61.2	32.8	6.0
Oils	0.61 (0.55–0.66)	0.62 (0.57–0.67)	55.1	35.3	9.6
Olives	0.59 (0.53–0.64)	0.6 (0.54–0.65)	66.1	24.3	9.6
Nuts and seeds	0.56 (0.5–0.62)	0.59 (0.53–0.64)	55.7	36.5	7.8
Sugar	0.74 (0.7–0.77)	0.72 (0.68–0.76)	57.9	33.9	8.3
Sweets	0.6 (0.54–0.65)	0.62 (0.56–0.67)	53.5	37.9	8.6
Sweetened drinks	0.72 (0.68–0.76)	0.72 (0.68–0.76)	57.0	37.0	6.0
Tea	0.66 (0.61–0.71)	0.71 (0.67–0.75)	54.8	37.1	8.1
Salty snacks	0.62 (0.57–0.67)	0.46 (0.38–0.53)	59.7	30.8	9.4
Salt	0.5 (0.43–0.56)	0.51 (0.43–0.57)	47.5	40.3	12.3

## Discussion

FFQs are commonly used in epidemiological studies to collect dietary information (4, 12, 13). While different FFQ designs—qualitative vs. quantitative or dish-based vs. item-based—have been used in various studies, the ultimate importance is for the FFQ to accurately capture what it was intended to measure so that diet-disease associations can be correctly made (14). In this study, we evaluated the validity and reproducibility of the PERSIAN Cohort FFQ in seven locations across Iran and found it to be appropriate to rank individuals based on their food group intake.

### Questionnaire design and administration

We designed this FFQ by modifying the validated GCS questionnaire, making it more concise and less detailed, as extensive FFQs lead to fatigue and decreased accuracy (15, 16). Also, given that a common error in self-reported questionnaires, including FFQs, is overestimation of foods consumed (5, 14, 17), and that inclusion of multiple foods or varieties of a food from the same group increase overestimation (5), we limited the number of food items in our FFQ, to foods with the highest frequency of consumption in our study population and only included enough detail to capture major dietary intakes and to avoid overlap between items. For example, the GCS questionnaire records chicken intake in ten separate items, distinguishing between various parts consumed, which makes reporting difficult and also may result in overlap and overestimation in the reported intakes; but we reduced the ten items to one item only, enquired about the overall frequency and amount of chicken intake. A direct comparison of correlations in chicken intake or other similar modifications between our FFQ and the GCS FFQ is not possible since we assessed food group intake and they evaluated nutrient intakes.

A similar comparison, however, may be made between the PERSIAN and the TLGS FFQs, which with 168 items, also recorded varieties of several foods. We asked about red meat use in one item—lamb or beef, as ground meat or cubes—while TLGS recorded beef, lamb and ground meat as three separate items. We reported red meat intake as a separate group in our analysis, while TLGS grouped all animal proteins together. Nonetheless, the DEA-SCC obtained in our study for *Red Meat* (0.52 to 0.59), *Chicken* (0.42 to 0.54), *Eggs* (0.46–0.54) and *Fish* (0.35–0.42) were higher than those reported for the TLGS *Meats* group (0.37–0.39 in men and 0.36–0.37 in women). Similar groupings of a single food item, or different items with similar nutrients were also performed throughout our FFQ. While this may decrease accuracy in the estimation of some nutrients, we believe that it limits overestimation of energy intake, while at the same time being easier for participants to report.

Another common problem seen with many dietary data collection methods, especially FFQs, is energy misreporting, most frequently seen as underreporting of nutrient-dense foods by participants (18, 19). Previous studies have found the following individuals to be most prone to underreport their intake: women, those with higher body mass index, lower literacy and education, as well as individuals of the lower socioeconomic status (18–22). While underreporting is sometimes intentional, especially by overweight/obese individuals, not all underreporting is intended, and participant fatigue, memory problems, as well as misperception of portion sizes can also lead to it (18). Strategies to limit underreporting have been suggested, some of which were used in our study. For example, we designed a shorter questionnaire compared to those previously validated to reduce participant fatigue and used common household measures, pictures and food models for a better estimation of portion sizes. Some interviewing techniques were also employed such as repeating participants’ responses back to them for various food items. Hearing their reported intake from the interviewers sometimes made participants realize they had misreported and corrected their responses. In addition, meal counting for grain intake was also used to limit under and over reporting of the most energy-contributing foods in the Iranian diet (described in greater detail in the following sections).

Our FFQ was interviewer-administered because some participants in smaller cities and villages were illiterate or with low education. But in general, interviewer-administered questionnaires result in systematically more desirable responses to lifestyle-related topics (23). In addition, interviewers trained on the same administration protocols can guide participants the same way and limit individual variations in interpretation of questions.

Interviewer-administered 24 h were chosen as the reference method in this study. Diet records, however, are considered more precise than 24 h and are suggested as the first reference method of choice in validation studies. This is so, because they share the least correlated errors with the FFQs, compared to other methods including the 24 h (4). For example, the FFQ relies on memory, whereas diet records do not, as foods are recorded at the same time they are consumed. Also, portion sizes are estimated when completing FFQs, but they are measured and exact amounts are written in diet records. The 24 h, on the other hand, shares these errors with the FFQ, and therefore its use as the reference method in validation studies yields to higher correlations that are a result of correlated errors. Nevertheless, the 24 h are most commonly used across validation studies due to their feasibility (24) and are considered the primary alternative to diet records, especially in instances when low participant cooperation/motivation for the completion of the diet records is expected or when participants have low literacy levels (4). In our study too, the 24 h seemed as the most reasonable option and most suitable for our population, given their low literacy levels (about 42% being illiterate of with only primary education). The USDA multiple-pass method was used to conduct the 24 h, which has been previously validated in different populations (25, 26).

### Validity

Our results showed that our FFQ is moderate-to-highly acceptable in estimating intakes of major energy-contributing food groups in the Iranian diet. The DEA-SCC between FFQ1, FFQ2, and FFQ1&2 vs. 24 h ranged from 0.23–0.7, 0.27–0.76 and 0.3–0.79, respectively, with most values being between 0.4–0.7 in all three comparisons. Previous validation studies of food group intakes have reported correlations between 0.3–0.8 (2, 4, 5, 13, 16, 27). To our knowledge, only the TLGS and the IHHP FFQs have been validated by assessing food group intakes in the Iranian population, however, the IHHP simplified FFQ, being focused on food habits related to cardiovascular diseases, is different in questionnaire design, foods included and validation groupings than the TLGS FFQ and ours, and therefore, its findings are not discussed in this manuscript. The median DEA-SCC observed by TLGS for FFQ1 and FFQ2 were 0.43 and 0.44 in men, and 0.43 and 0.37 in women, respectively, compared to the median DEA-SCC of 0.52 (FFQ1), 0.52 (FFQ2) and 0.58 (FFQ1&2) in our overall population (2).

We observed stronger SCC in food groups consumed at greater frequencies. The strongest correlations belonged to simple sugars, tea, grains, oils/fats, followed by dairy, vegetables, fruits, and animal proteins. Grains are the main staple foods of Iranians, used daily as bread and rice and for most individuals at every meal. We therefore placed great emphasis on the grains section of the FFQ and interviewing protocol. We ensured that all major grains consumed are included in the questionnaire and that local breads are also added, to not miss a major energy-contributing food item. Also, we tried to limit over/underreporting in grain consumption, by having the interviewers count the frequency of grain use per week based on the reported use of all grains, and enquire about patterns of grain use *if* over/under-reporting was observed. For example, if more than 21 uses of all grains combined was counted (the typical number of meals consumed/week), interviewers asked if grains are used in between meals as well, or if multiple types of grains are used simultaneously in one meal, to make sure over-reporting is limited. Likewise, if less than 21 meals were counted, interviewers asked participants if they routinely omit meals or not eat any grains at meals—not often customary with the Iranian cuisine—to make sure the amount recorded is not underreported. Necessary changes were then made, if needed. Therefore, we believe the correlations obtained in *Refined*/*Whole Grains* are closer to participants’ true intake than expected from an FFQ.

Tea consumption also showed strong correlations, because of its frequency of use, often drunk multiple times per day by most individuals. Interestingly, correlations of tea and sugar intake were very close, showing that the FFQ may also capture certain repetitive dietary habits, as many Iranians use sugar/sugar cubes daily to sweeten tea. The strongest correlations observed in TLGS also belonged to tea and sugar (2).

Correlations regarding solid fat and oil intake were also strong (0.65–0.78), given that they are also used predominantly daily in cooking. With the high rate of obesity and other NCDs related to high calorie and fat intake, these results are acceptable for use in future association studies. Our findings for fat intake differ from those observed in TLGS, where SCC ranged from 0.03–0.32 in men and 0.33–0.51 in women. Hosseini Esfahani et al. explained the weak associations observed in men, to be due to their lack of culinary knowledge, as women mostly cook in the Iranian culture (2). We tried to overcome this in our study by completing the questionnaire of spouses simultaneously. As explained, families enrolled in the PERSIAN Cohort on the same day and their FFQs were completed at the same time. Much emphasis was made on each individual reporting *their own* usual intake and spouses were not allowed to respond on behalf of one another except in the case of food items referred to as “hidden items” in the study protocol, such as salt, oil, tomato paste, etc. where the amount used in cooking is often not known by men who do not engage in cooking, and not visibly seen in their plate while eating. For these items, women reported the frequency and overall amount used in cooking, then each individual would report the portion of the total dish they would typically eat each time, and that proportion was used to estimate how much of the “hidden item” was consumed by each individual. This method may have influenced the stronger accuracy of fat/oil intake observed in our study.

Our FFQ was less valid at estimating *Legume* intake, with both C-SCC and DEA-SCC being below 0.3 in FFQ1 vs. 24 h and below 0.4 in the other two comparisons. *Other Meat*, *Pizza* and *Fresh Fruit Juice* also followed similar correlation patterns in the comparisons made. SCC related to legume intake was weak in TLGS as well (0.26–0.43 in men and 0.1–0.18 in women), possibly because legumes are mostly used in mixed dishes and stews in Persian cuisine, making their portion size difficult to report (2). The weak correlations observed in our study for *Other Meat*, *Pizza* and *Fresh Fruit Juice* were expected however, given their low median intake, ranging from 0 to 2.5 grams per day.

On average, 51–54% of individuals were classified correctly in the agreement analysis between the data collection methods. These findings are acceptable and compare to those observed by previous studies (2, 15, 28).

### Reproducibility

When assessing reproducibility, EA-ICC ranged from 0.42 to 0.72; correlations between 0.4–0.8 are typically seen in studies evaluating reproducibility of food group intake (4, 5, 29). Given that our second FFQ was administered one year after the first, real changes in dietary habits may have affected the lower correlations observed.

The complexity of a questionnaire also affects its reproducibility (30). Typically, questionnaires recording portion sizes tend to produce lower reproducibility due to higher variations in responses (5). Our FFQ, not only recorded portion sizes, but also gave individuals a choice for portion size reporting, using various tools, as they were also free to choose any time interval for the frequency of food consumption, not being limited by pre-determined frequency intervals. Therefore, our reproducibility results are more susceptible to random errors in comparison to qualitative FFQ or other, simpler methods.

Interestingly, foods groups with low median intake and weak validity, such as *Fresh Fruit Juice*, *Pizza* and *Other Meats*, had acceptable reproducibility, showing that they are consistently not eaten frequently in our study population and may possibly even be omitted from the FFQ in future uses.

### Strengths and limitations

- Perhaps one important strength of our study is the diversity of the study population. Our sample size exceeds typical recommendations for a validation study (between 100–200 individuals) (4). We exceeded this sample size not to increase precision—as increases over 200 do little for precision (4)—but to include an adequate number of individuals from each study location and have the diversity needed to use this FFQ in different Iranian populations.
- Repeating the 24 h twice monthly for a total of 24 records is another strength, trying to account for variations in foods consumed over one year.
- All interviewers were trained by the same individual and tools used for portion size estimation were centrally purchased and distributed to cohort centers to ensure consistency. The fact that our FFQ must be administered by an interviewer increases precision, while at the same time can be seen as a limitation because it may influence underreporting of foods perceived as unhealthy and over-reporting of healthy foods. It also adds to the personnel cost of studies wanting to use this questionnaire. But having a self-administered questionnaire was not possible in the PERSIAN Cohort due to a considerable proportion of the population having low literacy.
- Addition of the *local food items* (mostly breads and sweets) to the FFQ for each center is another strength of our questionnaire, making it appropriate for use in various populations of Iran by taking into account their different local foods and dietary habits. As previously described, grains (various breads and rice) are the staple food in Iran and the most energy-contributing foods, being consumed at all meals. And while the three main breads used across Iran (Lavash, Barbari and Sangak) were included in our questionnaire as *standard food items*, some areas included in the PERSIAN Cohort did not use any of these breads and not including the local breads would have led to inaccurate recording of their energy intake as no bread consumption would have been recorded. But in order to make sure all FFQs, despite the different local items, are analyzed the same, the *local food items* for each center were equated to the *standard items* by nutritionists, after data collection and therefore analyzed data from the FFQs in one PERSIAN Cohort site is not different from the others.
- We tried to limit biases in reporting by having the same interviewers who completed the cohort FFQs, complete the 24 h, using the same tools. This may have, on the other hand, caused an overestimation in correlations between methods, further increasing the correlated errors previously described.
- Correlations between FFQ2 and the 24 h were higher in comparison to those of FFQ1 and the 24 h. This was expected, however, as FFQ1 measured food intake 1 year prior to the start of the study, while the time of data collection in both FFQ2 and the 24 h coincided, both recording the intake of foods during the 1-year study period (the 24 h, recording food intake each month for one year, and FFQ2 recording food intake at the end of that same year, retrospectively). Another reason however for the higher correlations, may be that individuals had become more aware of their food intake during the study period, due to the monthly questionnaire completions and the fact that they knew they would have to complete another FFQ at the end of the study, and therefore it is possible that FFQ2 was actually completed with greater precision. This is an unavoidable limitation that is seen in validation study designs. We tried to provide better means of comparison for the validity and reproducibility evaluation of our questionnaire, however, by presenting correlations with FFQ1 and also with the mean of the two FFQs as well.
- Because our FFQ is shorter than those previously validated in Iran, a food item commonly consumed by a participant may have been included in the 24 h, but not the FFQ. Also, for food group or food item analysis, items recorded in the 24 h must be combined to correspond items on the FFQ, which adds sources of error (4).

## Conclusion

The PERSIAN Cohort FFQ is appropriate to rank individuals by their food group intake. Validity and reproducibility of the questionnaire in assessing dietary patterns and nutrient intakes must be further evaluated.

## Data availability statement

The raw data supporting the conclusions of this article will be made available by the authors, without undue reservation.

## Ethics statement

The studies involving human participants were reviewed and approved by Digestive Diseases Research Institute, Tehran University of Medical Sciences (IR.TUMS.DDRI.REC.1398.001). The patients/participants provided their written informed consent to participate in this study.

## Author contributions

SE, AH, HP, WW, and RM have contributed to the design of the research study. SE, HP, EF, RH, HH, MM, ZM, YP, and AD have contributed to study execution and data collection. SE and MS performed the data cleaning and statistical analysis, respectively. SE, AH, and MS prepared the manuscript. All other authors reviewed, commented on, and approved the final text.

## Funding

This study was supported by the Digestive Diseases Research Institute, Tehran University of Medical Sciences through Grant no. 97-03-37-39,212. The Iranian Ministry of Health and Medical Education has contributed to the funding used in the PERSIAN Cohort Study through Grant no. 700/534.

## Conflict of interest

The authors declare that the research was conducted in the absence of any commercial or financial relationships that could be construed as a potential conflict of interest.

## Publisher’s note

All claims expressed in this article are solely those of the authors and do not necessarily represent those of their affiliated organizations, or those of the publisher, the editors and the reviewers. Any product that may be evaluated in this article, or claim that may be made by its manufacturer, is not guaranteed or endorsed by the publisher.
