# Supplementary material for: Validity and reproducibility of a food frequency questionnaire assessing food group intake in the PERSIAN Cohort Study
Source: Front Nutr. 2023 Aug 4;10:1059870. doi: 10.3389/fnut.2023.1059870 (PMC10436288; doi:10.3389/fnut.2023.1059870)
Supplement: Supplementary file 1 [file Data_Sheet_1.pdf]

SUPPLEMENTARY MATERIAL DESCRIPTIONS

Gender-specific analysis of correlation coefficients regarding the validity and reproducibility of the PERSIAN Cohort FFQ are provided in the following three tables.

Abbreviations:

FFQ: Food Frequency Questionnaire

24 h: 24-hour dietary recall

Supplementary Table 1. Crude, energy-adjusted and de-attenuated energy-adjusted Spearman Correlation Coefficients comparing FFQ1, FFQ2 and mean of FFQ1 and FFQ2 with the 24 h in men									
Food Groups	FFQ1 vs. 24 h			FFQ2 vs. 24 h			FFQ1&2 vs. 24 h		
	Crude	Energy-Adjusted	De-attenuated energy - adjusted	Crude	Energy-Adjusted	De-attenuated energy-adjusted	Crude	Energy-Adjusted	De-attenuated energy-adjusted
Whole Grains	0.51	0.52	0.59	0.50	0.53	0.60	0.47	0.49	0.56
Refined Grains	0.72	0.60	0.81	0.69	0.51	0.68	0.76	0.60	0.81
Legumes	0.28	0.24	0.27	0.33	0.27	0.31	0.36	0.31	0.36
Fish	0.25	0.24	0.27	0.38	0.39	0.44	0.35	0.35	0.39
Red Meat	0.43	0.39	0.49	0.50	0.45	0.56	0.50	0.45	0.56
Processed Meat	0.43	0.43	0.5	0.44	0.45	0.53	0.46	0.47	0.55
Chicken	0.44	0.35	0.49	0.53	0.47	0.66	0.55	0.48	0.67
Eggs	0.42	0.37	0.45	0.46	0.44	0.53	0.49	0.45	0.55
Other Meat	0.23	0.22	0.25	0.26	0.24	0.28	0.26	0.25	0.29
Pizza	0.28	0.29	0.35	0.25	0.25	0.3	0.28	0.29	0.35
Cheese	0.46	0.47	0.56	0.53	0.54	0.65	0.54	0.57	0.68
Dairy	0.47	0.45	0.54	0.61	0.55	0.66	0.61	0.59	0.71
Vegetables	0.55	0.45	0.54	0.58	0.56	0.67	0.63	0.58	0.70
Fresh Fruit Juice	0.27	0.28	0.32	0.19	0.19	0.21	0.25	0.26	0.29
Fruit	0.41	0.45	0.56	0.43	0.51	0.63	0.46	0.54	0.67
Dried Fruit	0.39	0.42	0.52	0.37	0.36	0.44	0.41	0.42	0.52
Solid Fats	0.58	0.55	0.68	0.71	0.69	0.86	0.72	0.69	0.86
Oils	0.49	0.52	0.66	0.59	0.60	0.76	0.59	0.62	0.79
Olives	0.34	0.34	0.43	0.47	0.48	0.61	0.44	0.45	0.57
Nuts and Seeds	0.58	0.56	0.65	0.53	0.51	0.59	0.60	0.58	0.67
Sugar	0.68	0.66	0.85	0.68	0.62	0.80	0.74	0.73	0.94
Sweets	0.40	0.47	0.55	0.31	0.37	0.44	0.37	0.44	0.52
Sweetened Drinks	0.49	0.49	0.63	0.55	0.58	0.74	0.55	0.57	0.73
Tea	0.66	0.64	0.81	0.72	0.66	0.83	0.76	0.75	0.95
Salty Snacks	0.33	0.32	0.38	0.29	0.29	0.35	0.31	0.31	0.37
Salt	0.37	0.34	0.42	0.38	0.38	0.47	0.45	0.43	0.53

Supplementary Table 2. Crude, energy-adjusted and de-attenuated energy-adjusted Spearman Correlation Coefficients comparing FFQ1, FFQ2 and mean of FFQ1 and FFQ2 with the 24 h in women									
**Food Groups**	**FFQ1 vs. 24 h**			**FFQ2 vs. 24 h**			**FFQ1&2 vs. 24 h**		
	**Crude**	**Energy-Adjusted**	**De-attenuated energy - adjusted**	**Crude**	**Energy-Adjusted**	**De-attenuated energy-adjusted**	**Crude**	**Energy-Adjusted**	**De-attenuated energy-adjusted**
**Whole Grains**	0.53	0.50	0.57	0.52	0.50	0.57	0.54	0.52	0.59
**Refined Grains**	0.61	0.50	0.67	0.57	0.49	0.66	0.65	0.53	0.71
**Legumes**	0.25	0.23	0.26	0.39	0.37	0.42	0.34	0.33	0.38
**Fish**	0.34	0.35	0.39	0.33	0.35	0.39	0.37	0.38	0.43
**Red Meat**	0.45	0.46	0.57	0.50	0.51	0.63	0.50	0.51	0.63
**Processed Meat**	0.33	0.33	0.39	0.34	0.34	0.40	0.36	0.36	0.42
**Chicken**	0.41	0.36	0.51	0.48	0.43	0.60	0.51	0.46	0.65
**Eggs**	0.43	0.41	0.50	0.46	0.43	0.52	0.52	0.50	0.61
**Other Meat**	0.21	0.21	0.24	0.31	0.30	0.35	0.28	0.28	0.32
**Pizza**	0.25	0.25	0.30	0.33	0.34	0.41	0.31	0.32	0.39
**Cheese**	0.49	0.46	0.55	0.61	0.59	0.71	0.61	0.59	0.71
**Dairy**	0.51	0.46	0.56	0.55	0.55	0.66	0.61	0.58	0.7
**Vegetables**	0.49	0.39	0.47	0.49	0.50	0.60	0.57	0.53	0.64
**Fresh Fruit Juice**	0.26	0.27	0.30	0.31	0.30	0.34	0.32	0.33	0.37
**Fruit**	0.44	0.42	0.52	0.46	0.48	0.60	0.49	0.50	0.62
**Dried Fruit**	0.39	0.41	0.50	0.44	0.46	0.57	0.47	0.52	0.64
**Solid Fats**	0.62	0.60	0.75	0.69	0.68	0.85	0.73	0.71	0.88
**Oils**	0.58	0.58	0.74	0.71	0.70	0.89	0.70	0.69	0.88
**Olives**	0.35	0.36	0.46	0.39	0.40	0.51	0.38	0.39	0.50
**Nuts and Seeds**	0.51	0.49	0.57	0.46	0.42	0.49	0.51	0.48	0.56
**Sugar**	0.65	0.61	0.78	0.74	0.68	0.87	0.75	0.71	0.91
**Sweets**	0.37	0.40	0.47	0.38	0.40	0.47	0.39	0.42	0.50
**Sweetened Drinks**	0.50	0.50	0.64	0.50	0.50	0.64	0.52	0.52	0.67
**Tea**	0.68	0.62	0.78	0.74	0.66	0.83	0.78	0.71	0.89
**Salty Snacks**	0.32	0.31	0.37	0.32	0.30	0.36	0.35	0.34	0.41
**Salt**	0.36	0.29	0.36	0.43	0.34	0.42	0.47	0.37	0.46

**Supplementary Table 3. Reproducibility assessed by Intraclass Correlation Coefficients (ICC), comparing FFQ1 and FFQ2 in men and women**				
**Food Groups**	**Men**		**Women**	
	**Crude ICC (95% CI)**	**Energy-Adjusted ICC (95%CI)**	**Crude ICC (95% CI)**	**Energy-Adjusted ICC (95%CI)**
**Whole Grains**	0.61 (0.52-0.69)	0.67 (0.6-0.73)	0.64 (0.57-0.7)	0.62 (0.55-0.68)
**Refined Grains**	0.72 (0.66-0.78)	0.7 (0.63-0.76)	0.75 (0.7-0.79)	0.67 (0.61-0.73)
**Legumes**	0.45 (0.32-0.56)	0.43 (0.3-0.54)	0.38 (0.26-0.48)	0.41 (0.3-0.51)
**Fish**	0.55 (0.44-0.64)	0.54 (0.44-0.63)	0.56 (0.47-0.63)	0.52 (0.42-0.6)
**Red Meat**	0.6 (0.51-0.68)	0.61 (0.52-0.68)	0.5 (0.41-0.58)	0.59 (0.51-0.65)
**Processed Meat**	0.53 (0.42-0.62)	0.54 (0.43-0.62)	0.52 (0.43-0.6)	0.64 (0.57-0.7)
**Chicken**	0.69 (0.62-0.75)	0.61 (0.52-0.68)	0.7 (0.64-0.75)	0.67 (0.61-0.73)
**Eggs**	0.47 (0.35-0.57)	0.54 (0.43-0.63)	0.56 (0.48-0.63)	0.53 (0.44-0.6)
**Other Meat**	0.66 (0.59-0.73)	0.61 (0.52-0.68)	0.43 (0.32-0.52)	0.47 (0.36-0.55)
**Pizza**	0.61 (0.51-0.68)	0.7 (0.63-0.76)	0.49 (0.38-0.57)	0.49 (0.39-0.57)
**Cheese**	0.48 (0.36-0.58)	0.57 (0.47-0.65)	0.57 (0.48-0.64)	0.6 (0.53-0.67)
**Dairy**	0.74 (0.67-0.79)	0.69 (0.62-0.75)	0.64 (0.57-0.7)	0.65 (0.58-0.7)
**Vegetables**	0.73 (0.67-0.78)	0.66 (0.58-0.72)	0.54 (0.46-0.62)	0.55 (0.46-0.62)
**Fresh Fruit Juice**	0.35 (0.2-0.47)	0.39 (0.24-0.5)	0.43 (0.32-0.52)	0.57 (0.49-0.64)
**Fruit**	0.69 (0.62-0.75)	0.7 (0.63-0.76)	0.3 (0.56-0.69)	0.66 (0.6-0.72)
**Dried Fruit**	0.66 (0.58-0.73)	0.57 (0.47-0.65)	0.55 (0.47-0.62)	0.53 (0.44-0.61)
**Solid Fats**	0.66 (0.58-0.72)	0.62 (0.53-0.69)	0.68 (0.62-0.73)	0.72 (0.67-0.77)
**Oils**	0.62 (0.53-0.69)	0.62 (0.53-0.69)	0.6 (0.53-0.67)	0.62 (0.54-0.68)
**Olives**	0.6 (0.51-0.68)	0.63 (0.55-0.7)	0.58 (0.5-0.65)	0.58 (0.5-0.65)
**Nuts and Seeds**	0.61 (0.52-0.69)	0.61 (0.52-0.69)	0.52 (0.43-0.6)	0.58 (0.5-0.65)
**Sugar**	0.73 (0.66-0.78)	0.72 (0.66-0.78)	0.72 (0.68-0.77)	0.72 (0.67-0.77)
**Sweets**	0.56 (0.46-0.64)	0.6 (0.5-0.67)	0.62 (0.54-0.68)	0.64 (0.57-0.7)
**Sweetened Drinks**	0.74 (0.68-0.79)	0.74 (0.67-0.79)	0.63 (0.56-0.69)	0.69 (0.63-0.74)
**Tea**	0.62 (0.54-0.7)	0.59 (0.49-0.66)	0.68 (0.62-0.73)	0.76 (0.72-0.8)
**Salty Snacks**	0.81 (0.76-0.84)	0.71 (0.65-0.77)	0.4 (0.28-0.5)	0.35 (0.23-0.46)
**Salt**	0.55 (0.45-0.64)	0.48 (0.36-0.58)	0.46 (0.36-0.55)	0.47 (0.37-0.55)
